# The Treatment of Perforation in Typhoid Fever

**Published:** 1900-09-01

**Authors:** 


### Hospital Clinics and Medical Progress.

### THE TREATMENT OF PERFORATION IN TYPHOID FEVER.

A question which has been much discussed during recent years and to which attention has lately again been drawn is, what to do when symptoms of perforation occur in the course of typhoid fever. We say when symptoms arise, and we do so advisedly, for the history of cases in which operation has been done in the hope of closing a presumed perforation, and in which no perforation has been found, shows that the signs of this accident are not so absolutely distinctive as some writers have imagined. And this is where the real difficulty arises. If the question were merely what to do in cases in which perforation with extravasation had undoubtedly occurred, it would seem reasonable to hold that any operation which gave the slightest hope would be not only justifiable but obligatory. One has then always before one's mind the fact that the best of men have laid open the abdomen in full belief that they would find a perforation and have had to sew it up again without finding anything of the sort, so that the question is complicated by this reservation that we cannot always be sure of the condition we have to deal with. Granting, however, that the diagnosis is correct, a good deal of experience is accumulating in favour of opening the abdomen in such cases, and making an attempt either to close the perforation, or to drain the infected area.

The question has several times been discussed in the medical societies, and a not inconsiderable number of cases have been reported. In January of this year Dr. W. W. Keen reported in the Journal of the American Medical Association a series of 158 cases in which operation had been performed for perforation. More recently a report appeared in the Boston *Medical and Surgical Journal* for June 28th by Drs. G. B. Shattuck, J. Collins Warren, and Farrar Cobb upon 24 cases of typhoid fever operated on in consequence of the appearance of symptoms of peritoneal infection from perforation. These authors, besides confirming the fact that cases occur in which on operating nothing is found to account for the symptoms, make the very interesting observation that peritoneal infection in typhoid fever may arise from other causes than perforation. For example, cases have been met with in which, without any perforation having taken place, the necrotic bases of typhoid ulcers have given rise to a general septic peritonitis, while, again, the symptoms of perforation have been found to have been caused by a general septic peritonitis arising from a ruptured mesenteric gland. Another point worth noting is that in a considerable majority of the cases of perforation the primary disease had been of the type which one would describe as mild.

Another very important point, on the due recognition of which the success of operative interference will probably be found to depend to a large extent, is that in many of the cases definite premonitory symptoms had been observed some time before the time of the actual extravasation. It would, indeed, appear as if one would have to give up the old idea that perforation is an unforeseeable accident, which may come unaware upon anyone, and that rather one must be always on the look-out, not perhaps for the classical symptoms of perforation so much as for the premonitory symptom which shows that it is likely to occur, a symptom which probably indicates the occurrence of a small perforation and a limited peritonitis, preceding by some hours the complete rupture and extravasation. This warning symptom is spoken of as pain in the abdomen, more or less localised in the right inguinal region, a pain sometimes preceding the severe symptoms by hours or even by days, and it is insisted that such localised pain is not common in typhoid fever apart from peritoneal infection.

It would seem indeed that perforation by no means always produces its classical symptoms. In the Medico-Chirurgical Transactions, Vol. LXXX., a case is reported in which Mr. Bowlby operated successfully for a perforating typhoid ulcer, and a consideration of the symptoms in that case shows well how far from typical may be the symptoms even of a considerable extravasation, and it is stated in the report that, from the condition found, it seemed probable that the aperture in the bowels had existed for some time before having been temporarily but insecurely closed by adhesion. The American cases show the same thing, viz., that a local peritoneal infection may occur some time before the onset of the severe symptoms. Turning now to the results of operative treatment, these reports work out to a mortality of something between 19 and 24 per cent. The numbers, however, are as yet too small to enable one to speak seriously of percentages, especially in view of the great variety of conditions met with. But it seems clear that, if the observations in regard to premonitory symptoms should be confirmed, and if by any other method it should become possible to diagnose peritoneal infection with certainty before the occurrence of complete extravasation, the surgeon might be able to intervene with still greater hope of success than is the case at present.

